# Leveraging a Consumer-Based Product to Develop a Cancer-Specific Mobile Meditation App: Prototype Development Study

**DOI:** 10.2196/32458

**Published:** 2022-01-14

**Authors:** Jennifer Huberty, Nishat Bhuiyan, Taylor Neher, Lynda Joeman, Ruben Mesa, Linda Larkey

**Affiliations:** 1 Calm San Francisco, CA United States; 2 College of Health Solutions Arizona State University Phoenix, AZ United States; 3 Research Consultancy Little Rock, AR United States; 4 Lynda Joeman Research Consultancy Tonbridge United Kingdom; 5 Mays Cancer Center University of Texas Health San Antonio MD Anderson Cancer Center San Antonio, TX United States; 6 College of Nursing and Health Innovation Arizona State University Phoenix, AZ United States

**Keywords:** cancer patients/survivors, meditation, mHealth, app development, qualitative research

## Abstract

**Background:**

Mobile meditation apps may offer a long-term, accessible, and effective solution for ongoing symptom management in cancer patients/survivors. However, there are currently no commercial cancer-specific meditation apps that reflect cancer specialist expertise, input from cancer patients/survivors, and features and content specific to cancer patients’/survivors’ needs.

**Objective:**

The aim of this study was to gain insight (via surveys, daily journals, and focus groups) from cancer patients/survivors, health care providers, and current subscribers of Calm (a consumer-based mobile meditation app) who were patients/survivors to develop a prototype of a mobile meditation app specifically designed for cancer patients/survivors.

**Methods:**

Participants were recruited via prior partnerships, word-of-mouth referrals, and recruitment posts on Facebook and Instagram. Cancer patients/survivors and health care providers were instructed to download and use the Calm app for at least 10 minutes a day for 7 days, complete an online daily journal for 7 days, and participate in a virtual focus group (one for cancer patients/survivors and one for providers). Current Calm subscribers who were cancer patients/survivors completed an online survey about different aspects of the Calm app and participated in a third virtual focus group. Data were qualitatively analyzed using a combination of deductive and inductive coding.

**Results:**

A total of 27 participants (11 cancer patients/survivors, 10 health care providers, 6 current Calm subscribers) completed the study. Similar themes and subthemes were found across surveys, daily journals, and focus groups, and fell into two major categories, content and functionality, with cancer-specific and noncancer-specific themes identified within each category. The majority of content preferences and suggestions that arose were cancer-specific, such as content related to negative emotions or feelings (eg, anxiety, grief, trauma/posttraumatic stress disorder, fear of recurrence, isolation), positive feelings and finding meaning (eg, gratitude, storytelling, acceptance), scenarios and experiences (eg, waiting, treatment-specific mediations), type and stage of cancer journey, and movement modifications. Some of the noncancer-specific themes under app content included sleep, music, and visualizations. In terms of app functionality, the majority of participants expressed interest in having a section/tab/area of the app that was specifically geared toward cancer patients/survivors. Preferences and suggestions for cancer-specific functionality features included options based on symptoms or journey, being able to communicate with other patients or survivors to share suggestions for specific meditations, and having an emergency toolkit for patients/survivors.

**Conclusions:**

Findings from cancer patients/survivors, health care providers, and current Calm subscribers who were patients/survivors to be incorporated into the development of the prototype fell into two major categories: (1) content of the app and (2) functionality of the app. The prototype’s form and function will be pilot-tested among 30 cancer patients/survivors in a 4-week study, and the resulting feasibility data will be used to inform the final app design and an efficacy study.

## Introduction

The chronic symptom burden among cancer patients/survivors is debilitating and costly. Many survivors have ongoing symptoms that can last for 10 or more years that severely affect their quality of life, ability to return to work, and independence [1]. Long-term, accessible, and effective solutions for ongoing symptom management for the ~15.5 million US cancer patients/survivors are sorely needed as the chronic symptom burden costs the health care system an estimated US $125 billion annually and impacts the economy through US $115 billion in lost worker productivity [2,3].

Meditation has been shown to improve symptoms such as pain, sleep disturbance, anxiety, and fatigue in cancer patients and survivors [4-7]. Such programs are often delivered in person, usually at a cancer center. However, these programs are expensive and difficult to sustain. Some patients struggle to attend even short-term programs at specialized clinics due to distance, scheduling, and symptom burden [8]. There is a need to translate beneficial meditation interventions into more accessible and sustainable formats.

Mobile apps can provide effective, accessible meditation instruction to cancer patients/survivors. Mobile apps and online meditation programs have demonstrated short-term benefits for cancer patients/survivors [9,10]. Positive effects have been reported from even 10 minutes of daily meditation practice [11-13], and research suggests that the therapeutic benefits of brief daily sessions can be seen in as little as 4 weeks [9,10]. In one study, 97.0% (318/328) of cancer patients had access to a smartphone and were willing to use app-based meditation [9,10].

Cancer-specific meditation apps are needed to ensure clinical acceptability, effectiveness, and safety for patients/survivors. Research shows that targeting specific patient groups allows for wider reach, higher adherence rates, and greater impacts on health behaviors [14,15]. To improve the targeting, uptake, and long-term impact of eHealth interventions in patient populations, the Center for eHealth Research and Disease Management recommends obtaining patient user and health care provider feedback in the early design phase [16]. Although cancer patients/survivors have reported that meditation apps targeting the general population are useful [9], there are important differences in cancer patients’ physical, emotional, and social needs that are not met by a general meditation app. These needs are incorporated into in-person meditation programs in cancer centers, because they are led by cancer experts who can modify the program according to the symptom burden and cancer-specific psychological states and experiences, and because they include the shared experiences of other cancer patients facing the challenge of meditating [10,17-19]. There is a need for such tailoring into commercial mobile apps. A standalone cancer-specific app that reflects cancer specialist expertise, input from cancer patients/survivors, and provides features and content specific to the unique physical and psychological needs of cancer patients/survivors is urgently needed.

Of the 150 apps marketed for cancer to date, there are no evidence-based, commercially available meditation apps for cancer patients/survivors [17,20]. A systematic review [19] identified only two app-delivered meditation studies, both focusing on breast cancer, and ultimately neither program aimed for commercialization. Most app companies lack the scientific expertise needed to develop such tools or lack access to clinical populations for tailoring the app to their needs. There is a dearth of evidence-based cancer-specific apps because behavioral researchers have worked in isolation, and once evaluated in a clinical trial, no meditation apps have reached commercialization [19]. Although technological solutions hold promise for accessible, low-cost, and scalable approaches to meditation delivery, many studies have shown that long-term engagement with standard commercial apps is very low [21]. Companies make profits for the app purchase and subscriptions but are not incentivized to deliver effective strategies to measurably improve outcomes; this is particularly the case for specialized populations. To address these barriers to developing, testing, and eventually making available an evidence-based meditation app for the millions of cancer patients and survivors, behavioral scientists at Arizona State University, cancer care providers at Mays Cancer Center, and designers at Calm (a meditation app company) have partnered to accelerate the integration of effective techniques for patients’/survivors’ long-term symptom-management needs.

Calm is the top-grossing health and fitness app in the United States, with over 100 million downloads and 4 million paying subscribers. Calm is subscription-based, and has over 220 guided meditations to teach users the basics of meditation, how to incorporate meditation into one’s life, and programs for intermediate and advanced meditators [9,10]. Calm effectively teaches the skills and practice of meditation, using generic meditations to address various sources of stress (eg, work, relationships) experienced by the general population. Research suggests that Calm can reduce stress and increase mindfulness, and its use is also associated with incremental increases in mental and physical health, stress, and sleep in the general population [22,23]. Calm is also an effective short-term meditation program to reduce the symptom burden among cancer patients/survivors [10]. Based on the clinical needs of cancer patients/survivors and the lack of standalone commercialized cancer-specific apps, the purpose of this project was to gain insight (via surveys, daily journals, and focus groups) from cancer patients/survivors, health care providers, and current Calm subscribers who were patients/survivors to develop a prototype of a mobile meditation app for cancer patients/survivors. We here describe how our findings from surveys, daily journals, and focus groups will be used to develop content for and produce the cancer-specific meditation app prototype.

## Methods

### Participants

This study was approved by a university-affiliated institutional review board. Potential participants were cancer patients/survivors (diagnosed within the last 3 years), health care providers (physicians, oncology nurses, cancer care coordinators, directors of integrative programs in cancer centers, and not-for-profit partners), and current subscribers to the Calm app who were cancer patients/survivors (diagnosed within the last 3 years).

### Participant Recruitment

Cancer patients/survivors were recruited via word-of-mouth referrals by cancer care physicians who had previously partnered with the investigative team (eg, from the Mays Cancer Center at UT Health San Antonio, MD Anderson; the Mayo Clinic in Scottsdale, AZ, and Rochester, NY; University of Arizona; Wake Forest; Leukemia and Lymphoma Society; and the American Cancer Society). The investigative team recruited health care providers by compiling a list of providers who had diverse backgrounds in cancer care (nursing, social work, physician), cancer types, and patient demographics, along with their contact information. Cancer patients/survivors who were referred to the team and health care providers from the list were then sent an email that included a brief study description and asked if they were interested in participating in the study. Current Calm subscribers who were patients/survivors were recruited via recruitment posts on the Calm Community Facebook and Instagram pages. The recruitment posts included a brief study description and a link to the online eligibility screening survey.

### Eligibility and Consent

All interested potential participants were directed to complete an online eligibility screening survey (via REDCap). See Textbox 1 for inclusion criteria.

Eligible participants (cancer patients/survivors, health care providers, and current Calm subscribers who were patients/survivors) were sent a link to an online informed consent document (via REDCap) with details about the study. All participants provided consent via an electronic signature prior to participating in the study.

**Eligibility criteria for inclusion in the study.**

**Patients/survivors**

- Cancer diagnosis within past 3 years
- Own a mobile smartphone (iPhone with iOS 9.0 or later or Android 4.1 or later)
- Willing to download a mobile app
- Able to read and understand English

**Health care providers**

- Physicians, oncology nurses, cancer care coordinators, directors of integrative programs in cancer centers, or not-for-profit partners
- Own a mobile smartphone (iPhone with iOS 9.0 or later or Android 4.1 or later)
- Willing to download a mobile app
- Able to read and understand English

**Current Calm subscribers**

- Cancer diagnosis within past 3 years
- Currently have a subscription to and uses Calm
- Own a mobile smartphone (iPhone with iOS 9.0 or later or Android 4.1 or later)
- Willing to download a mobile app
- Able to read and understand English

### Study Procedures

Cancer patients/survivors and health care providers completed a brief online survey assessing the birth date, cancer patient/survivor or health care provider status, current meditative practice, and current or prior use of the Calm app. These participants were then sent instructions on how to download the Calm app on their smartphone and instructed to (1) use the Calm app for at least 10 minutes a day for 7 days, (2) explore the content and features of the app, and (3) complete an online daily journal (via RedCAP) for 7 days. The daily journal included (1) time of day and time spent using Calm; (2) responses to app options and what motivated them to make certain choices; (3) their experiences during the guided meditations; (4) ideas about life experiences of a cancer patient to include in a prototype; (5) responses to prompts related to strategies from social cognitive theory, such as “In what ways might the content or features of the app support a sense of social modeling from other cancer patients or providers?” and “How might goal setting and rewards be incorporated appropriately?”; and (6) if not a cancer patient or survivor, to reflect upon the perspective of those they treat or serve. For the purpose of this analysis, only the qualitative answers (3-6) were included as they were formative responses to the app’s development. The link to the online journal was sent to participants daily during the 7-day period. After using the Calm app for 7 days and completing the daily journal, cancer patients/survivors and health care providers were emailed to schedule a date and time to participate in a virtual focus group.

Current Calm subscribers who were patients/survivors were directed to complete an investigator-developed online survey assessing demographics; what they currently liked and disliked about the Calm app; which Calm series, instructors, and components they found helpful or unhelpful; and their suggestions for cancer-specific adaptations/modifications to the Calm app. At the end of the survey, current Calm subscribers who were patients/survivors were given the opportunity to provide their email address to be entered into a drawing to win one of 10 Magic of Sleep books from Calm and were asked if they wanted to opt-in to participate in a virtual focus group. Those who expressed interest in participating in the focus group were emailed to schedule a date and time.

### Focus Groups

Three total focus groups were conducted: one with cancer patients/survivors, one with health care providers, and a third with current Calm subscribers who were patients/survivors. The focus groups were conducted by a member of the research team, were held virtually using Zoom teleconferencing software, and took approximately 1 hour to complete. Focus groups explored specific domains of design: acceptability, demand, practicality, adaptation, and integration (ie, open-ended experiential and cancer-referenced content explorations). See Multimedia Appendix 1 for the specific focus group questions. Three app developers from Calm participated in the focus groups for practical guidance and to meet target needs in the prototype design (only participating in the groups to develop the app and not for data purposes). Prior to participating, the developer signed a confidentiality agreement. The focus groups were audio- and video-recorded. The audio recordings from the focus groups were sent to a professional, Health Insurance Portability and Accountability Act–compliant transcription company (Landmark Associates, Inc, Phoenix, AZ) to be transcribed. Transcripts were used for qualitative data analysis.

### Qualitative Analysis

The focus group transcripts were imported into NVivo 12 qualitative analysis software (QSR International) and the survey responses and daily journal text files were imported into MAXQDA qualitative analysis software (VERBI Software) for the purpose of coding and analysis. The research investigators developed a codebook to be used in the qualitative analyses. Using the codebook, one investigator independently coded the surveys, a second investigator independently coded the daily journals, and a third qualitative analyst independently coded the focus group transcripts. All qualitative analyses followed the recommended approaches of Braun and Clarke [24] and Swain [25], and a combination of deductive and inductive coding was used to analyze the data. Top-level categories and themes were identified deductively based on the main information requirements of the study and the categories of issues covered in the surveys, daily journals, and focus group discussions. Emergent themes were identified inductively from the transcripts and text files. The coding and analysis process was iterative, with identified themes and subthemes being continually reviewed and revised until these were felt to most accurately represent the expressed experiences and views of the participants and also to allow for comparability of findings between the focus groups (ie, patients/survivors, health care providers, and current Calm subscribers). Findings are summarized using verbatim quotes to illustrate these themes and subthemes and to ensure that these views and experiences are conveyed in the voices of the participants themselves.

## Results

### Demographic Characteristics

A total of 27 participants (11 cancer patients/survivors, 10 health care providers, and 6 current Calm subscribers who were patients/survivors) completed the surveys or daily journals and participated in the focus groups. Participant characteristics can be found in Table 1. These sample sizes were chosen and deemed appropriate based on previous qualitative research studies utilizing similar methodology [26-29].

The themes and subthemes identified in the surveys, daily journals, and focus groups are summarized in Table 2, and are described more in-depth below.

**Table 1:** Descriptive characteristics of the focus group participants.

Characteristic		Cancer patients/survivors (n=11)	Health care providers (n=10)	Current Calm subscribers (n=6)
Age (years), mean (SD)		47.7 (13.1)	46.7 (6.6)	50.5 (12.2)
**Gender, n (%)**				
	Female	9 (82)	8 (80)	6 (100)
	Male	1 (9)	1 (10)	0 (0)
	Prefer not to answer	1 (9)	1 (10)	0 (0)
**Race, n (%)**				
	White, European-American, or Caucasian	9 (82)	8 (80)	5 (83)
	Black or African American	1 (9)	0 (0)	0 (0)
	Asian or Asian American	0 (0)	1 (10)	0 (0)
	Arab or non-Arab North African/Middle Eastern	0 (0)	0 (0)	1 (17)
	Prefer not to answer	1 (9)	1 (10)	0 (0)
**Ethnicity, n (%)**				
	Hispanic or Latino	1 (9)	0 (0)	0 (0)
	Not Hispanic or Latino	9 (82)	9 (90)	6 (100)
	Prefer not to answer	1 (9)	1 (10)	0 (0)
**Education, n (%)**				
	Employed	7 (64)	9 (90)	5 (83)
	Unemployed/unable to work	1 (9)	0 (0)	1 (17)
	Homemaker	1 (9)	0 (0)	0 (0)
	Retired	1 (9)	0 (0)	0 (0)
	Prefer not to answer	1 (9)	1 (10)	0 (0)
**Annual income (US $), n (%)**				
	≤50,000	0 (0)	0 (0)	0 (0)
	50,000-74,999	1 (9)	0 (0)	1 (17)
	74,000-99,999	2 (18)	1 (10)	0 (0)
	>100,000	7 (64)	7 (70)	4 (67)
	Prefer not to answer	1 (9)	2 (20)	1 (17)
**Education, n (%)**				
	≤Associate/2-year degree	0 (0)	0 (0)	0 (0)
	Associate/2-year degree	1 (9)	0 (0)	0 (0)
	Bachelor's degree	6 (55)	3 (30)	3 (50)
	Graduate school or above	3 (27)	6 (60)	3 (50)
	Prefer not to answer	1 (9)	1 (10)	0 (0)
**Cancer type, n (%)**				
	Breast	4 (36)	N/A^a^	4 (67)
	Ovarian	2 (18)	N/A	0 (0)
	Endometrial	1 (9)	N/A	0 (0)
	Colon	1 (9)	N/A	0 (0)
	Rectal	1 (9)	N/A	0 (0)
	Blood	2 (18)	N/A	1 (17)
	Jaw	0 (0)	N/A	1 (17)
**Currently undergoing cancer treatment, n (%)**				
	Yes	7 (64)	N/A	4 (67)
	No	4 (36)	N/A	2 (33)
**Current mindful practice, n (%)**				
	Yes	4 (36)	6 (60)	6 (60)
	No	7 (64)	4 (40)	0 (0)
Mindful practice (days/week), mean (SD)		5 (2.5)	3.3 (2.9)	5 (5.2)
**Current Calm use, n (%)**				
	Yes	4 (36)	3 (30)	6 (60)
	No	7 (64)	7 (70)	0 (0)
Calm use (days/week), mean (SD)		4 (4.2)	1.1 (0.9)	5 (5.2)

^a^N/A: not applicable.

**Table 2:** Overall themes and subthemes identified from participants’ preferences and suggestions for app adaptations and modifications for a cancer-specific meditation app.

Themes and subthemes				Surveys		Daily journals					Focus groups				
				Current Calm users		Patients/ survivors		Health care providers		Current Calm users			Patients/ survivors		Health care Providers
**App content**															
	**Cancer-specific**														
		Gratitude			✓		✓		✓			✓		✓	
		Grief			✓							✓			
		Waiting			✓		✓					✓		✓	
		Type and stage of cancer journey			✓		✓					✓		✓	
		Movement modifications^a^			✓							✓			
		Self-care	✓						✓						
		Pain^a^	✓		✓		✓					✓			
		Nausea			✓		✓								
		Storytelling	✓		✓		✓		✓			✓		✓	
		Acceptance	✓		✓		✓								
		Anxiety	✓		✓		✓		✓			✓		✓	
		PTSD^b^/trauma	✓												
		Fear of recurrence	✓											✓	
		Isolation	✓		✓							✓			
		Perspective	✓		✓										
		Treatment-specific meditations^a^	✓						✓					✓	
		Breathing based meditations	✓		✓										
		Positive feelings and finding meaning^a^							✓			✓		✓	
		Negative emotions or feelings^a^							✓			✓		✓	
	**Noncancer-specific**														
		Sleep	✓		✓		✓								
		Music			✓		✓								
		Visualizations			✓		✓								
**App functionality**															
	Community features						✓		✓			✓		✓	
	Scheduling/tracking		✓		✓										
	More options to personalize		✓		✓		✓		✓			✓		✓	
	Cancer-specific vs noncancer-specific		✓		✓		✓					✓			
	**App navigation**														
		Suggest specific options to others	✓		✓				✓			✓		✓	
		Algorithm that provides options based on symptoms or journey	✓		✓		✓								
		Searching for topics	✓		✓									✓	
	New users/meditators		✓		✓		✓								
	Guide to the app		✓		✓		✓							✓	
	Accessibility for disabilities				✓										
	Emergency toolkit				✓										
	Terminology													✓	
	Accessible to families/caregivers		✓		✓									✓	

^a^In the analyses of the focus group data, these indicated subthemes were categorized as themes. See Textbox 2 for details regarding which subthemes were linked to these overall themes in the focus group analyses.

^b^PTSD: posttraumatic stress disorder.

### Surveys

The overall completion rate for the surveys was 100% (eg, all 6 current Calm subscribers who were patients/survivors submitted an online survey). The completion rate for the questions in the survey ranged from 92% to 100% (12/13 to 13/13 questions completed) for the demographic questions and from 70% to 100% (7/10 to 10/10 questions completed) for the questions assessing Calm preferences and potential adaptations/modifications. Survey responses fell into two major categories: (1) content of the app and (2) functionality of the app. Themes and subthemes identified in the surveys are represented in Table 2, and full illustrative verbatim quotes from the surveys representing each theme and subtheme can be found in Multimedia Appendix 2.

### Journals

Completion rates for the daily journals varied from the providers and patients/survivors with 77.1% (54/70) of the providers’ daily journals being completed and 87.0% (67/77) of the patients’/survivors’ journals completed. However, in the provider group, only 53.4% (187/350) of the questions were answered during the 7 days the daily journal was administered compared to 66.9% (206/308) of the questions being answered by the patients/survivors group. Daily journals fell into two major categories, content of the app and functionality of the app, and within each category, themes that were cancer-specific and noncancer-specific emerged. Themes and subthemes identified in the daily journals (completed by patients/survivors and health care providers) are represented in Table 2, and full illustrative verbatim quotes from the daily journals representing each theme and subtheme can be found in Multimedia Appendix 3 (cancer patients/survivors) and Multimedia Appendix 4 (health care providers).

### Focus Groups

Findings from the focus groups fell into two major categories: (1) content of the app and (2) functionality of the app. These themes are categorized under patients/survivors focus group, health care provider focus group, and current Calm subscriber focus group in Textbox 2, and a written description of each theme/subtheme follows.

**Focus group themes and subthemes.**

**Cancer patients/survivors**

Content themes (subthemes)

- Negative emotions or feelings (anxiety, fear, grief)
- Specific situations or experiences (isolation, waiting)
- Positive feelings and finding meaning (storytelling, gratitude)
- Types and stages of cancer
- Pain or physical discomfort
- Movement modifications

Functionality themes (subthemes)

- Overall focus and features (cancer-specific or not, community features, personalization)
- App navigation (having options suggested)

**Health care providers**

Content themes (subthemes)

- Negative emotions or feelings (anxiety, fear)
- Specific situations or experiences (treatment-specific, waiting)
- Positive feelings and finding meaning (storytelling)
- Stages of the journey

Functionality themes (subthemes)

- Overall focus and features (options and personalization, community features, terminology, accessible to family or caregivers)
- App navigation (guide to the app, emergency toolkit, search facility)

**Current Calm subscribers**

Content themes (subthemes)

- Negative emotions or feelings (anxiety)
- Specific situations or experiences (treatment-specific, waiting)
- Positive feelings and finding meaning (gratitude, self-care, storytelling)

Functionality themes (subthemes)

- Overall focus and features (options and personalization, community features)
- App navigation (having options suggested)

### Patient/Survivor Focus Groups

#### Content of the App

##### Negative Emotions or Feelings

Participants identified a range of negative emotions or feelings that they had experienced during their cancer journeys, which they felt that the Calm app might help with. The most commonly cited types of emotions were anxiety and fear.

There is still that fear and anxiety every time I go back, for—for 3 months, 3 months, 3 months, 3 months, and then it was 6—now it's 6 months. And I'm like, “What if I missed something?” What if I missed something? Every little symptom is, like, oh, my gosh. What now?

Helping cancer patients deal with feelings of anger and grief about what they feel they have lost in terms of health or body parts that were removed was also seen as an important function of the Calm app. Some participants highlighted the risk of triggering negative reactions in cancer patients, and expressed a preference for generic content rather than content focused specifically on cancer patients.

##### Situations or Experiences

Various experiences associated with the cancer journey were identified; in particular, the app is seen to be helpful for the experiences of isolation and waiting.

Several participants explained the ways in which they had experienced forms of isolation while undergoing cancer treatment, as a result of having to give up work and avoidance of usual social interactions because of their treatment schedule or symptoms. A sense of isolation was often felt particularly by those with rare forms of cancer who struggled to find others with whom to share their experiences: *“*Cancer is very isolating. People cut themselves off from others and it’s hard to figure out how to deal with other people.”

The participants also stressed the anxiety often associated with periods of waiting for appointments or test results, and suggested that perhaps the app might include content relevant to typical milestones in the cancer journey and the emotions associated with these.

##### Positive Feelings and Finding Meaning

Many of the focus group participants reported that they felt reassured or encouraged by hearing the stories of others, especially cancer survivors. They stressed that it was the sense of connection with the actual storytellers based on shared or relatable experiences that was most appealing, and were less keen on having celebrities narrate their stories.

When you hear it come from another patient, there is a different level of, you know—um, you know, it…it is authentic. It is…it is insightful. It is beyond what someone that might have a canned speech or a celebrity might even say.

The focus group participants also discussed the importance to them of content that helped to process their emotions and accept or come to terms with their cancer experience, without imposing pressure to feel a particular way: “If the app could help you process, sort of like therapy, frankly…what you’re dealing with and what you…what you bring out of it without, um, telling you, you have to be happy.”

For some, learning to feel gratitude for what they value in life was an important aspect.

##### Types and Stages of Cancer

The focus group discussion highlighted the ways in which the Calm app can be helpful for individuals at different stages of their cancer journey or with different forms of cancer. For example, participants contrasted the anxiety of a new cancer diagnosis with the ongoing stress of long-term or rare forms of cancer. The ongoing effects on individuals even after successful treatment were also highlighted.

##### Pain or Physical Discomfort

Two particular aspects of pain or discomfort that the app might potentially help with were discussed: the need for movement modifications due to pain or surgery and the experience of hot flashes.

I just had surgery, um, 4 weeks ago today, um, and I have to sleep in a new position with, like, compression garments on, and it’s very uncomfortable, um, and it takes me a long time to fall asleep because I'm not getting enough exercise. Um, and so I think that the—that was something that I took from the Calm app that I didn’t really—um, hadn’t really done in other, like, meditation practices.

A meditation for hot flashes, but anything that would help everybody who gets that—like you said, we’re all in the same room.

##### Movement Modifications

It was stressed that the app content should take into account any mobility restrictions that users might be subject to.

I think it needs to be tailored to those people that may not, you know, be—they have—they’re compromised as far as what they can do, uh, to the Calm Body side of—like, without makin’ ‘em feel bad, so to speak, you know.

#### App Functionality

##### Overall Focus and Features

###### Cancer-specific or Not

The participants discussed whether the content of the app should be specifically tailored to cancer patients or be more generic, and a range of views were expressed. Some highlighted the benefits of having cancer-specific content that patients could relate to: “I think that it would be really helpful to be able to tailor it to cancer patients and some of those fears and*—*and anxieties that are inherent, I think, to*—*to cancer patients in general.”

Others expressed the view that some experiences of cancer patients are similar to those of individuals with other conditions or life circumstances, and that more general content should therefore be included to appeal to a wider population of users.

It could be accessible and relevant to a lot of different people, but that theme of—of loss and grieving what you wish you had and finding a place of acceptance with this is what I have and I’m grateful for what I have, um, or I can make my peace with it, or just accepting it for today is enough to get me to the next day, and then to the next week, and moving on.

It was suggested that achieving an appropriate balance between cancer-specific and more general content would be helpful, especially to meet the needs of cancer patients themselves and to meet different psychological needs at various stages of their journey.

I think you would want to have it tailored enough that it was relevant to a cancer patient, but also broad enough that it felt relevant on the days when you’re 13 days out of chemo and you've still got 6 more days and it’s of just kind of a regular day.

###### Community Features

Most participants indicated that they would be in favor of a community area on the app where users could interact and post comments. Some explained that they would feel reassured by knowing that others share and understand their own experiences in ways that other people cannot, regardless of the specific type of cancer they have.

I can’t do all the things I would like to do, you know, as—as a mother and all, but I realize there’s other people there with—with my same problem and my, you know, same issues and my same concerns, but I no longer feel alone, like there's something wrong with me ‘cause there’s other people that are in the same boat with me. And so I think those stories are really important.

However, one participant argued that they would not be in favor of a community feature as any cancer-specific content would be likely to trigger negative emotions: “I like the app because it’s not social media, and I want to not be triggered by the cancer content, whether it’s, like, knowingly or unknowingly.”

###### Personalization

Some indicated that they would like to be able to personalize the app to their own circumstances, or to have the option of monitoring their heart rate before and after meditating.

If you could just put some of your own notes or tags that are personal to you, um, I thought that might have been a helpful way that if I was looking back a month later, thinking, “Why was I so frustrated that day?” or “Why was I anxious that day?” to be able to give yourself those tags to help give it context.

When you do the check-in kind of thing, I thought if you, um, gave the option to the person to record the heart rate.

##### App Navigation

Several participants suggested that they would appreciate being able to see suggestions or recommendations for particular meditations, or to have options suggested for them depending on how they felt or what they were dealing with at the time.

Many were in favor of having a search facility for meditations on particular topics, with some indicating that it can be hard to find what they need in the current version of the app: “it’d be helpful if you had a way of finding these topics that were on the cancer patient’s mind, either by filters or topics, subjects.”

It was stressed, however, that descriptions and terminology need to be carefully considered to enable cancer patients to find suitable content but without triggering negative emotions.

the words are important, and we have to find words in the cancer scenario that wouldn’t trigger the anxiety of people…it can be, you know, anxiety over appearance, over deformities due to cancer, over certain parts of the body…So the words are important, and…we wanna be able to give people the option of not having certain triggers.

Participants also stressed the importance of ensuring that the app is accessible for use by the many cancer patients who also have disabilities such as sight or hearing disorders.

### Provider Focus Groups

#### Content of the App

##### Negative Emotions or Feelings

The provider focus group participants indicated they felt their patients would mostly find the Calm app beneficial for dealing with feelings of anxiety or fear: “I think people would use it during treatment, either when they’re sitting in an infusion chair, when they’re waiting for a scan—there needs to be a scanziety section to this somehow.”

##### Specific Situations or Experiences

The participants also identified waiting for and undergoing treatment as situations in which patients might benefit from using the app, along with experiences such as insomnia.

##### Positive Feelings and Finding Meaning

Similar to the patients and user participants, many of the providers indicated that cancer patients can find it reassuring or helpful to hear the stories of others who have gone through or survived similar experiences. Similar to the patient participants, the health care providers also expressed the view that the authentic shared experience is more important than having a celebrity narrator.

I don’t think it matters as—as long as the person is, um, is, you know, kind of breaking that fourth wall and saying, “I’ve had dark days, too,” or, “I’ve been there. I have a similar experience.” Doesn’t matter if it’s a celebrity or the guy next door. I think people will—will connect with that.

One of the participants indicated that the app might be more helpful for individuals who have completed their treatment and are trying to process and make sense of what they have been through. Another stressed its potential importance in helping to provide hope at earlier stages of the cancer journey.

##### Stages of the Journey

It was suggested that the app might usefully act as a type of roadmap to the experiences and stages involved in a cancer diagnosis and journey, helping patients feel less overwhelmed and providing support with the various experiences involved.

A cancer patient’s journey is really complicated and overwhelming. And so one thing that I thought of is, you know, having a roadmap as you get into cancer is really helpful, and having different sorts of meditations through chemo, surgery, radiation, the waiting room, infusion, et cetera.

#### App Functionality

##### Overall Focus and Features

The provider participants reported that they liked the variety and different lengths of meditations available on the Calm app, as well as the range of narrators and voice tones, which were felt to meet the different needs and preferences of their patients: “I think different people resonate with different types of meditations in different voices, so I actually think a variety is really nice to have.”

##### Community Feature

Most of the provider participants indicated that they would be in favor of a community area on the app where users could interact and post comments, highlighting the perceived benefits to patients of connecting with those going through similar experiences. Like some of those in the users group, however, they also acknowledged the potential risks of this that need to be managed.

I think you have to be really careful because, like, even in facilitating support groups, right, you have those people who kind of take over and you—or you have to—you have to have kind of very strict guidelines, um, of what they, you know, what they can share…I kinda see it more like a support group, that there’s gotta be rules and kind of very strict guidelines of what is said.

##### Terminology

Several of the provider participants discussed the importance of using the right terminology to attract a range of people, and noted that the term “meditation” has negative connotations for some. They suggested using more neutral terms such as symptom management, or terminology relating to specific situations such as sleep or stress reduction.

One time, a patient was asking me what she could do to, you know, help herself, and I said, “Well, some people believe in meditation,” and she said to me, “Oh, I don’t meditate. I pray.” You know? So sometimes, the word “meditation” for a certain patient, um, means something that they don’t believe in, and so I feel like sometimes I have to be very careful when I recommend something like that, ’cause pe—people get the wrong idea.

##### Accessible to Family or Caregivers

Some suggested that the app should also be designed for use by the family or carers of cancer patients.

##### App Navigation

###### Guide to the App

Many of the provider participants argued that the current app can be overwhelming and confusing for many cancer patients, and suggested that it would be beneficial to have a simple step-by step guide or roadmap for how to use it.

Having some sort of roadmap of either a landing page for a first-time user or almost an aerial view of Calm and the different pieces of it, or, like, a 1-minute tour of Calm. You know, something that a person can press on really quickly to just get their bearings.

One recommended that the guide might take the form of a video of a real person such as a cancer survivor to provide a sense of personal connection.

It was also suggested that the guide might provide options based on how the user indicated they were feeling at the time.

###### Emergency Toolkit

Some participants suggested that it would be helpful for the app to be designed at least in part as a type of “emergency toolkit” that would allow patients to quickly identify resources helpful for dealing with particular needs or situations. This was conceptualized by some as “symptom management.”

I almost wonder, like, if you logged in, what would you like to address today, like, symptom management versus stress. Like, I’m feeling nauseous, I’m feeling, you know, I have anxiety about, you know, I have to lay on this—on the radiation table for 15 minutes and I need something that’s 15 minutes long, or, you know, I need something—I’m waiting for my test results back from the doctor that I can just put in. Like, you almost wonder if you could have a symptom checklist because it’s, you almost need, like, an emergency toolkit and then you almost need, like, your day-to-day stuff.

###### Search Facility

One participant expressed the view that patients with specific requirements from the app would appreciate the inclusion of a search facility and that this would help reduce a sense of overwhelm from all the options available.

### Calm Subscribers Focus Group

#### Content of the App

##### Negative Emotions or Feelings

The user focus group participants identified in particular the importance of the Calm app in helping them deal with anxiety during treatment or when waiting for test results.

Some of my chemos were, like, 6, 7 hours. That’s a long time to be in a chemo chair, and I would listen to podcasts and I’d get really bored, especially, like, during COVID ‘cause they were all in isolation. Um, so I was doing a lot of Calm stuff ‘cause my anxiety would go up and down.

##### Specific Situations or Experiences

Various experiences associated with the cancer journey that the app is seen to be helpful for were identified, including undergoing treatment or waiting for test results, but also dealing with the experience of being a survivor and the continuing uncertainties that this can involve.

Once you’re done with chemo, then what? And I think that's where…just the fear of, you know, every 3 months you have to have scans and what’s that gonna show and trying to be brave through that. So I think, maybe, that should be part of, you know, maybe, a Calm, you know, app for cancer is not just while you’re in it but the—the next steps as well ‘cause it does—as we all know, it doesn’t just end. You know, it’s—it’s here with us.

One participant stressed that they prefer the meditations that relate to concrete daily experiences rather than those that were more abstract or general. Suggestions were also made for specific types of content, including healing visualizations and meditations suitable for teenage patients.

##### Positive Feelings and Finding Meaning

Many of the focus group participants indicated that they would like content that helped them process their experiences and develop a sense of positivity or acceptance. Meditations that helped them focus, be mindful, or feel a general sense of gratitude in their lives were regarded as helpful in this respect. Some participants emphasized the importance of self-care and self-compassion in the process of coming to terms with their diagnosis and coping with their treatments, and found the meditations that supported these types of feelings particularly helpful.

We’re so trained to go, go, go, go, go, take care of other people, it’s really hard to retrain myself that right now, my job is to heal and take care of myself and that that’s okay, and that it’s okay to slow down. It’s okay to lay down. It’s okay to have bad days, and we’re not—that’s not a mentality that we’re used to hearing and that—that’s cultivated in our society. So that’s been very helpful for me, the self-compassion one.

Some participants also mentioned that they would welcome the inclusion of educational snippets or stories of interest to them or relevant to their own experiences, such as content narrated by survivors, other cancer patients, or doctors.

It could be doctors. It could be nurses talking. It could be cancer survivors. It could be people talking about, like, what—what should be in a chemo bag. You know? I mean, typical little tidbit-type things of, you know, 1- or 2-minute wisdom of how to cope. It could patients telling their stories, uh, u-, up-lifting stories, um, but practical wisdom, that type of thing. I think that would be—I know I would love to hear that.

#### App Functionality

##### Overall Focus and Features

###### Options and Personalization

Some participants indicated that they would like to be able to personalize the app to suit their personal preferences, for example by varying the volume or voices of narrators or the background music. One also mentioned that they would like the option of longer guided meditations.

I would love to have, um, some of the longer guided ones. You know, have the option of doing a 10 or a 20 minute. I’ve recently found where I can set—I can do independent ones that are longer, and I just found that option, but, um, I would love a little bit of the guided ones to be longer as well.

###### Community Features

The participants in the user focus group were split in their views about whether the app should include a community feature where users could interact and post comments. Like many of those in the patient group, several explained that they would like to be able to connect with people going through similar experiences.

Although broadly in favor of a community feature, some highlighted that there can be risks involved in interacting with other cancer patients, such as increasing anxiety.

When you don’t hear from them for a while, like, the brain starts to go in directions that you don’t want it to go. So there’s—there’s a little bit of a balance that has to be managed here because of the anxiety that you already have and then anxiety that you’re—you may, sort of, take on because you’re worried about other folks that are managing their cancer.

Other participants were firmly opposed to the idea of having a community feature to the app, indicating that they would have no interest in using this feature or that would be off-putting to them: “That would be a very big negative for me, and it would be a drawback. It’s not what I want in an app. I don’t wanna social group.”

##### App Navigation

Some participants suggested that they would appreciate being able to see suggestions or recommendations for particular meditations, and to have these grouped together within themes using an autoplay facility.

I’d end up, like, endlessly tryin’ to scroll through tryin’ to find the stuff that I wanted to. If there was some way that they could loop or, um, go from one—like if there was series of, um, like, okay, if you’re a cancer patient doing treatment, here’s, like, a block of something that might be relevant for you. And maybe they can loop together.

Some of those in the user group were also in favor of having a search facility for meditations on particular topics, and indicated that it can be hard to find what they need in the current version of the app.

## Discussion

### Principal Findings

The purpose of this project was to gain insight (via surveys, daily journals, and focus groups) from cancer patients/survivors, health care providers, and current Calm subscribers who were patients/survivors to develop a prototype of a mobile meditation app for cancer patients/survivors. Findings from the surveys, daily journals, and focus groups fell into two major categories, content and functionality, and within these, themes that were cancer-specific and noncancer-specific. The major themes and subthemes that emerged within these categories will be integrated into the development and design of the cancer-specific meditation app prototype.

This study included both cancer patients/survivors who currently had paid subscriptions to the Calm app and cancer patients/survivors who did not have any prior experience using the Calm app. Although these differing experiences (ie, prior experience with the Calm app vs no prior experience with the Calm app) were expected to shape the participants’ responses to the survey, daily journal, and focus group questions, we found that the majority of the emergent themes and subthemes were consistent across these groups. Our findings suggest that prior use or familiarity with the current Calm app did not make a major difference in cancer patients’/survivors’ preferences and suggestions for a cancer-specific meditation app prototype.

All three groups (cancer patients/survivors, health care providers, and current Calm subscribers who were patients/survivors) shared themes that were both cancer-specific and noncancer-specific. Overall, the majority of content preferences and suggestions that arose were cancer-specific, such as content related to negative emotions or feelings (eg, anxiety, grief, trauma/posttraumatic stress disorder [PTSD], fear of recurrence, isolation), positive feelings and finding meaning (eg, gratitude, storytelling, acceptance), scenarios and experiences (eg, waiting, treatment-specific mediations), type and stage of cancer journey, and movement modifications. Similarly, in terms of app functionality, the majority of participants (including cancer patients/survivors, health care providers, and current Calm subscribers who were patients/survivors) expressed interest in having a section/tab/area of the app that was specifically geared toward cancer patients/survivors. Some preferences and suggestions for cancer-specific functionality features included an algorithm that provides options based on symptoms or journey, being able to suggest specific mediations to others or receive suggestions from other patients/survivors, and having an emergency toolkit for patients/survivors. Some of the noncancer-specific themes fell under the content of the app category, such as sleep content, music, and visualizations. Participants expressed that these content suggestions would not only be helpful to cancer patients/providers but could also be helpful to individuals without cancer.

Interestingly, some participants expressed that they wanted to be able to use the app without being reminded of their cancer and some indicated that they did not want to use this app as a tool to connect with other cancer patients/survivors. Given these findings, the final meditation app prototype will likely include a majority of cancer-specific content and functionality features but may also include more general content and features that are not cancer-specific.

Overall, the themes and subthemes that emerged in this study confirm the app content and functionality features found in previous research with cancer patients/survivors. For example, prior studies have found that cancer patients/survivors also experience more daily stressors than the general population that current apps fail to address, and need specific content on managing grief, PTSD symptoms, trauma, fear of death, pain, positivity, strong emotions, worry, life after cancer, body positivity, and others, in relation to meditation practice [21]. Other prior research found that due to the prevalence and burden of symptoms, cancer patients/survivors need to be aware of improvements in symptoms that accompany meditation and that further reinforce continued meditation practice [20]. A similar finding was reinforced in this study, in which participants suggested that an algorithm that provides options based on symptoms and being able to track symptoms would be functionality features that would be helpful for cancer patients/survivors. Finally, support from other patients/survivors specific to the challenges and benefits of meditation practice is also needed to help patients/survivors adapt their practice to their clinical manifestations, and cancer patients need brief calming meditations for very specific contexts (eg, before doctor visits or during chemotherapy). In these emergency scenarios, meditations that acknowledge the context and heightened stress are needed. Overall, many of our results reinforced or were in line with findings from previous studies assessing mobile app preferences and needs of cancer patients/survivors. In addition, we found similar themes across our three groups: the cancer patients/survivors, health care providers, and current Calm subscribers who were patients/survivors. Thus, these study findings will allow us to develop a mobile meditation app prototype that is targeted toward and can specifically meet the needs of cancer patients/survivors.

A future direction for this research is to finalize the development of the cancer-specific meditation app prototype, which is currently under development. The investigative team will work in partnership with developers from Calm to integrate the overall findings from this study to rapidly develop a server-side application programming interface (API) and an app for iOS. Themes and feedback from the surveys, daily journals, and focus groups will be used to extend the current Calm design, APIs, and app, so that the content, layout, and features in the app will address specific cancer-related long-term needs.

We will then beta test the feasibility of the cancer-specific meditation app prototype with a sample of cancer patients/survivors (N=30). We will recruit cancer patients/survivors with varying cancer types and stages, race/ethnicity, rural/urban geographic settings, and genders via social media and partnerships with nonprofit organizations. Beta-test participants will be asked to use the prototype for 4 weeks for at least 10 minutes per day, but will be encouraged to use it as much as they would like, mimicking how a new paying member would use the existing Calm app. Participation (meditations completed, time of day, length of time, components used) will be tracked over the 4 weeks. To determine acceptability, demand, and practicality, participants will be asked to complete weekly satisfaction questionnaires in addition to a final overall study satisfaction questionnaire immediately postintervention. In addition to a satisfaction questionnaire, we will ask the participants to volunteer for a semistructured interview to determine, using Bowen’s feasibility model [30], acceptability, demand, practicality, adaptation, and integration to optimize potential for uptake, engagement, and continued use at recommended levels (ie, at least 10 minutes a day). Themes and findings from the interviews will be used to translate findings into app-relevant recommendations (ie, further revisions if necessary) for efficacy testing. This approach is similar to methods used in other studies designing and developing mobile apps [31,32].

### Strengths and Limitations

This study has a number of strengths. The biggest strength of this study is the inclusion of three groups: cancer patients/survivors, health care providers, and current Calm subscribers who were patients/survivors. We were able to gain insight from end users at both the individual and clinic levels. We had contributions from those who will use the app and those who will prescribe the app. This is in alignment with the call from health service managers who recommend integrating accessible, complementary treatments into regular medical practice [33]. Another strength of this study was leveraging a popular consumer-based app (ie, Calm) to increase the potential success of the cancer-specific app. Calm has a large reach (~4 million paying users) and is a household name, which is likely to help the cancer-specific meditation app be successful on the market [34]. Additionally, when marketed to cancer care clinics, professionals, and patients/survivors, Calm may have the ability to offer subscriptions at highly reduced rates.

There are some study limitations to note. One study limitation was the lack of double coding during the qualitative analyses, as we did not have a second individual independently code the surveys, daily journals, or focus groups. Instead, one author (NB) coded the surveys, another author (TN) coded the daily journals, and a third author (LJ) coded the focus group transcripts. However, we found similar themes and subthemes across surveys, daily journals, and focus groups. Another limitation of the study was the limited generalizability, given that the majority of the sample was female, non-Hispanic White, and of relatively high socioeconomic status. Therefore, the results may not represent the views of male, racial/ethnic minority, and/or low-income populations. However, we did have broad representation of cancer type. The next step of this formative research is to pilot-test the app prototype among a sample of 30 cancer patients/survivors, and to address the current limitations in generalizability, we plan to recruit a more diverse population to participate in the pilot study.

### Conclusions

Cancer patients/survivors, health care providers, and current Calm subscribers who were patients/survivors completed surveys, daily journals, and focus groups to provide insight into the development of a cancer-specific mobile meditation app prototype. Findings fell into two major categories, content of the app and functionality of the app, and will be incorporated into the development of the prototype. The prototype’s form and function will be pilot-tested among 30 cancer patients/survivors in a 4-week study, and the resulting feasibility data will be used to inform the final app design and an efficacy study.

## Appendix

Multimedia Appendix 1 Focus group questions.

Multimedia Appendix 2 Themes, subthemes, and illustrative quotes from current Calm subscribers’ surveys.

Multimedia Appendix 3 Themes, subthemes, and illustrative quotes from cancer patients'/survivors’ daily journals.

Multimedia Appendix 4 Themes, subthemes, and illustrative quotes from health care providers’ daily journals.

## Abbreviations

API: application programming interface
PTSD: posttraumatic stress disorder
